# TA-Unet: Integrating Triplet Attention Module for Drivable Road Region Segmentation

**DOI:** 10.3390/s22124438

**Published:** 2022-06-12

**Authors:** Sijia Li, Furkat Sultonov, Qingshan Ye, Yong Bai, Jun-Hyun Park, Chilsig Yang, Minseok Song, Sungwoo Koo, Jae-Mo Kang

**Affiliations:** 1 Department of Artificial Intelligence, Kyungpook National University, Daegu 41566, Korea; lisijia@knu.ac.kr (S.L.); furkatsultonov@knu.ac.kr (F.S.); wnsgus126@knu.ac.kr (J.-H.P.); 2 Department of Information and Communication Engineering, Hainan University, Haikou 570100, China; sam970520@163.com (Q.Y.); bai@hainanu.edu.cn (Y.B.); 3 METROTECH Co., Ltd., Yeonam Bldg, 6, Yeongdong-daero 118-gil, Gangnam-gu, Seoul 06089, Korea; csyang@wjis.co.kr (C.Y.); sms@wjis.co.kr (M.S.); swkoo91@wjis.co.kr (S.K.)

**Keywords:** U-Net, triplet attention module, TA-Unet, road feasible domain segmentation

## Abstract

Road segmentation has been one of the leading research areas in the realm of autonomous driving cars due to the possible benefits autonomous vehicles can offer. Significant reduction of crashes, greater independence for the people with disabilities, and reduced traffic congestion on the roads are some of the vivid examples of them. Considering the importance of self-driving cars, it is vital to develop models that can accurately segment drivable regions of roads. The recent advances in the area of deep learning have presented effective methods and techniques to tackle road segmentation tasks effectively. However, the results of most of them are not satisfactory for implementing them into practice. To tackle this issue, in this paper, we propose a novel model, dubbed as TA-Unet, that is able to produce quality drivable road region segmentation maps. The proposed model incorporates a triplet attention module into the encoding stage of the U-Net network to compute attention weights through the triplet branch structure. Additionally, to overcome the class-imbalance problem, we experiment on different loss functions, and confirm that using a mixed loss function leads to a boost in performance. To validate the performance and efficiency of the proposed method, we adopt the publicly available UAS dataset, and compare its results to the framework of the dataset and also to four state-of-the-art segmentation models. Extensive experiments demonstrate that the proposed TA-Unet outperforms baseline methods both in terms of pixel accuracy and mIoU, with 98.74% and 97.41%, respectively. Finally, the proposed method yields clearer segmentation maps on different sample sets compared to other baseline methods.

## 1. Introduction

The self-driving car (also known as an autonomous vehicle) has been rapidly developing around the world. Since a self-driving car can automatically sense and understand its surroundings, and navigate a vehicle without human intervention, it has led to a growing number of manufacturers and researchers willing to invest significant efforts in this research area [1]. Additionally, technology can increase the factor of safe driving, and therefore reduce or avoid human errors in the driving process. Although self-driving cars are becoming increasingly important, there is still plenty of room for further development of related technologies.

It is important for the autonomous vehicles to be aware of their surroundings before they make a decision [2]. Thus, road segmentation is crucial in self-driving areas, which relates to recognizing the road conditions. Road segmentation can become extremely challenging at different times of day and weather conditions. Some recently proposed computer vision methods based on convolutional neural networks (CNNs) [3,4,5,6] can efficiently solve the segmentation problem [7,8,9,10,11]. In the realm of self-driving cars, the PLARD framework addresses the limitations of gaps in space for road detection, and improves road detection performance [12]. In the RNE-RoadSeg article, a new module, called surface normal estimator, is introduced, that leads to a boost in performance [13]. These methods are capable of demonstrating superior performance to humans. However, improved accuracy is gained by expanding the depth of CNNs, which, in turn, increases the time to train these state-of-the-art models [14,15,16]. Therefore, those state-of-the-art networks requiring enormous resources are not suitable for deploying them into practice. In comparison, U-Net has a great advantage in terms of parameter size, and achieves quality results in binary segmentation problems [17]. Additionally, inspired by the quality results of the U-Net model for the biomedical image segmentation task, there has been an increasing number of new methods that incorporate the U-shaped encoder–decoder architecture of the U-Net model, along with recently introduced techniques to achieve improved results in the semantic segmentation research area. Mixer U-Net is a method to solve automatic road extraction from UAV imagery [18]. Dense U-net employs DenseNet blocks in place of regular layers to achieve quality results in brain tumor detection tasks [19]. Furthermore, Residual U-net utilizes residual connections within each layer of both encoder and decoder parts of the network for the retinal vessel segmentation task [20]. Finally, uncertainty quantification (UQ) methods have been increasingly exploited in the field of autonomous driving as they play a key role in reducing uncertainty in optimization and decision-making processes [21,22].

Here, we adopt the U-Net network as a foundation because of its symmetric skip-connection feature [17]. The advantage of skip-connection is that it combines low-level feature maps with high-level ones. The spatial information not only can help to improve the precision of pixel-level location but also can spread and gather context information in high-level feature maps to low-level. However, the U-Net architecture has the following two critical problems: firstly, the network structure is too simple, and the result would be inaccurate in the segmentation process. Secondly, downsampling method in the network, i.e., max-pooling operation, collapses the feature map and leads to the loss of edge information.

To increase the complexity of the network and to achieve improved results by doing so, a growing number of attention modules are being exploited in computer vision research. In 2018, there was a famous article called Attention U-Net, where the authors added the attention module to the U-Net architecture [17]. Specifically, the attention gate was introduced to filter the propagated features through the skip connections before being concatenated with the mirroring decoder stage input. Adding attention modules into the traditional CNNs can improve the enhancement of the relevant regions which, in turn, boosts accuracy. However, this method can also cause a large parameter overhead.

In this paper, we adopt a novel architecture, dubbed TA-Unet, which is based on U-Net and injects triplet attention mechanism in the encoder layer [23]. The motivation for using triplet attention for road segmentation is twofold. Firstly, combining the existing framework and an attention U-Net in a proper way can improve the performance. Furthermore, the aim of triplet attention is to calculate the attention weights by capturing cross-dimensional interactions using the triplet branch structure, which makes it effective in road segmentation scenarios without adding too many parameters. The main contributions of this paper are summarized as follows:

1. We demonstrate the implementation of triplet attention in a standard U-Net architecture (TA-Unet) and apply it to the drivable road area segmentation task.
2. Compared to the state-of-the-art SGSN model provided by the UAS dataset, our model has significantly improved the mIoU and the accuracy rate.
3. Compared to the original FCNs (fully convolutional networks) for semantic segmentation, DANet (dual-attention network) for scene segmentation, and Attention U-Net, we have remarkably reduced parameter size while improving mIoU and accuracy [7,24].

The remainder of this paper is organized as follows. In Section 2 and Section 3 we introduce related works and the proposed TA-Unet in detail, respectively. Next, we present the experiments, results, and discussion in Section 4. Finally, the conclusion and future work are provided in Section 5.

## 2. Related Work

### 2.1. U-Net

U-Net is a classical algorithm for image segmentation using fully convolution networks [8]. The network was originally designed for solving problems in biomedical images, but since the results are good, it has been widely used in various areas of semantic segmentation, such as satellite image segmentation and road segmentation. The salient feature of U-Net-like networks is the symmetric skip connections which merge low-level feature maps of the encoder with high-level feature maps of the decoder. The spatial information that contributes to pixel-level localization accuracy is propagated from the low-level feature maps and aggregated into high-level contextual information. At each stage of the encoder, two 3×3 convolutional layers and ReLu activation function are applied, and then a 2×2 max-pooling layer is adopted to downsample the formed feature maps [25]. In the decoder part, the output of the encoder is first upsampled by deconvolution operation, and then the resulted output is concatenated with the mirroring encoder stage output before being processed with two 3×3 convolutional layers and ReLU activation function. Finally, every time the feature maps are downsampled by the max-pooling operation, some edge features are bound to be lost, and the lost features cannot be recovered from the upsampling operation. Therefore, in order to retrieve the lost edge features, a feature stitching method is exploited in the original U-Net [25].

### 2.2. Attention U-Net

Attention is widely applied in the task of text recognition of complex scenes, and the aim is to focus on digits to be recognized. Wei et al. proposed an end-to-end self-driving network that incorporates a sparse attention module. The model automatically attends to the most important regions within an image, which leads to the remarkable reduction in computation, and improves the planner safety [26]. In the Attention U-Net paper, soft attention is used in a CNN for medical images for the first time, and this module can replace hard attention in classification tasks and localization modules in organ localization tasks. The essence of the attention module is to enhance regions of interest while suppressing certain non-interest regions [27]. Compared to the original U-Net paper, the addition of attention mechanism can lead to a remarkable improvement in the accuracy rate of image segmentation. However, this approach results in significant computational overhead, so we are inspired by the Attention U-Net model, which successfully introduces attention mechanism into the U-Net network. Specifically, we adopt a novel attention mechanism which reduces the computational cost while improving the accuracy [28].

### 2.3. Triplet Attention

Triplet attention is one of the recently proposed methods that compute attention weights by capturing cross-dimensional interactions through the triplet branch structure [23]. The traditional technique to calculate channel attention includes first calculating weights and then using these weights to uniformly scale these feature maps. However, it is important to note that this approach requires the input tensor to be spatially decomposed into a single pixel by global average pooling in order to determine the weights for these channels. Since there is no interdependence between channel dimension and spatial dimension upon computing attention on a single pixel channel, it might lead to a large loss of spatial information [29,30]. Thus, the cross-dimension interaction concept has been introduced in the triplet attention mechanism, which enables to alleviate the spatial information loss problem by capturing the interaction between spatial dimension and input tensor channel dimension. Here, cross-dimensional interactions in triplet attention are introduced by capturing the dependencies between the (C,H), (C,W), and (H,W) dimensions of the input tensor through three branches, individually.

## 3. TA-Unet

In this section, we first introduce the core unit in triplet attention, and then explain the architecture of the proposed TA-Unet in detail.

**Figure 1:**
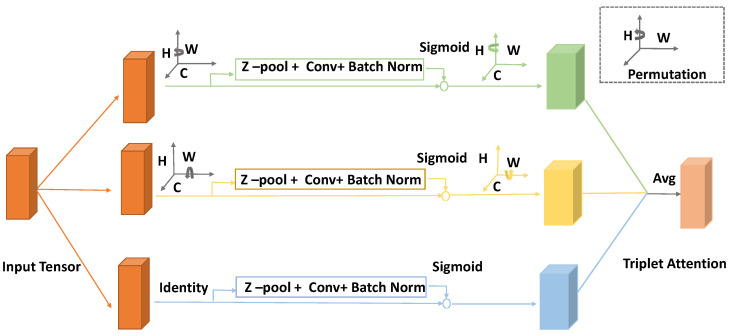
Detailed architecture of the triplet attention mechanism, which calculates attention weights based on a three-branch structure to capture cross-dimensional interactions. The first branch (green) computes channel dimension *C* and spatial dimension *W*, the second branch (yellow) captures channel dimension *C* and spatial dimension *H*, and the third branch (blue) computes spatial dependencies between *H* and *W*. The final output is an average of the resultant feature maps of the branches.

The goal of the attention mechanism is to focus on the key information and discard other parts within an image. One of the pioneering studies that utilized attention mechanism along with convolution operations was carried out in SENet, and it focuses only on the attention mechanism of the channel dimension [30]. In the successive CBAM model, the space and channel dimensions are emphasized, but they are computed separately and are computationally heavy [29]. However, in the triplet attention, dependencies are established between dimensions. Specifically, cross-dimension interactions are established through three branches to capture dependencies between the (C,H), (C,W), and (H,W) dimensions of the input tensor, respectively. It addresses the shortcomings of the previous studies by capturing the interaction between the spatial dimensions and the channel dimension of the input tensor with a negligible computational overhead. Figure 1 demonstrates the flowchart of the proposed triplet attention mechanism.

As the flowchart highlights, the triplet attention mechanism is composed of multiple parallel branches. The first branch computes attention weights across the channel dimension *C* and the spatial dimension *W*, while the second branch is responsible for *C* and *H*, and the final branch captures spatial dependencies across *H* and *W* [23]. The shape of the resultant outputs of all the branches are the same. In order to obtain the final output of the triplet attention mechanism, we simply take the average of sum of the individual branch outputs. Further, in order to calculate the channel attention, we exploit singular weights, which is considered a lightweight and efficient method. Specifically, the operation is performed by inputting scalars for each channel in the tensor and then using the singular weights to scale these feature maps uniformly. In practice, however, these singular weights are computed by spatially decomposing the input tensor into one pixel per channel via a global average pooling which leads to a significant loss of spatial information [23]. The authors of triplet attention have introduced a spatial attention module as a complementary method to address the attention of individual pixel channels. In fact, spatial attention focuses on the location in the channel, and channel attention aims to focus on the channel, allowing interaction between the channel dimension and the spatial dimension, as expressed by the dependencies between the (C,H), (C,W), and (H,W) dimensions of the input tensor, respectively. By concatenating the outputs of the average pooling and max pooling operations in the 0th dimension of the input, *Z-pool* reduces it to the 2nd dimension It has the advantage that the layer retains the actual rich tensor and reduces its depth while being lighter to compute. The following is the mathematical expression of the *Z-pool* operation:

(1) $$Z-pool\left(X\right)=[MaxPool_{0d}\left(T\right),AvgPool_{0d}\left(T\right)]$$

where $T\in R^{C\times H\times W}$ represents the output of a convolutional layer, and *C*, *H*, and *W* stand for the channels of the tensor or the numbers of filters, height, and width of the spatial feature maps, respectively. In addition to that, 0d is the 0th dimension across which max pooling and average pooling operations are performed. For a tensor of shape (C×W×H), the *Z-pool* operation results in a tensor of shape 2×W×H, which retains a rich representation of the actual tensor, while shrinking its depth.

As the name denotes, triplet attention is composed of three separate branches. For each branch, the shape of the output is the same as that of the input tensor. Given an input tensor $T\in R^{C\times H\times W}$, in the first branch, the input is rotated $90^{\circ }$ counterclockwise along the *H*-axis to make interactions between the height dimension and the channel dimension (H,W), which results in the shape of the input tensor (W×H×C). Furthermore, the resultant feature map is passed through the *Z-pool* to make it a (2×H×C)-shaped tensor. The next step is to convolve the formed feature map with a standard convolution layer followed by a batch normalization operation. The result of these operations is an intermediate output of (1×H×C) dimensions. A sigmoid activation function is performed on the output to obtain the attention weights. Finally, the resultant output $\hat{T_{1}}$ is rotated $90^{\circ }$ clockwise along the *H*-axis to keep it consistent with the input shape.

In the second branch, interactions between channel dimension and width dimension (C,W) are built. The first step is to rotate the *W* axis $90^{\circ }$ anticlockwise to obtain the shape (H×C×W). Then, the resultant output is processed through the *Z-pool* to form a (2×C×W) tensor. Similarly, as in the first branch, the output of the *Z-pool* operation is convolved through a standard convolution layer following a batch normalization operation to obtain (1×C×W). Subsequently, the obtained attention weights are then passed through a sigmoid activation layer. Finally, the resultant tensor is rotated $90^{\circ }$ clockwise along the *W* axis to retain the same shape as input *T*.

Unlike in the previous branches, in the third branch, we do not perform rotation operation. *Z-pool* is carried out to reduce channels of the input tensor *T* into two. The formed tensor $\hat{T_{3}}$ is further convolved by a standard convolution layer of kernel size k×k followed by a batch normalization layer. The resultant output is passed through a sigmoid activation function, and the output is a tensor of the shape (1×C×W). The resultant tensors of shape (C×H×W) of each branch of the module are then aggregated by averaging.

Given an input tensor $T\in R^{H\times C\times W}$, the process of attaining a refined feature map *S* from the triple attention mechanism can be expressed by the following equation:

(2) $$S=\frac{1}{3}\bar{(\hat{T_{1}}\sigma \left(\psi_{1}\left({\hat{T^{*}}}_{1}\right)\right)}+\frac{1}{3}\bar{(\hat{T_{2}}\sigma \left(\psi_{2}\left({\hat{T^{*}}}_{2}\right)\right)}+T\sigma (\psi_{3}\left(\hat{T_{3}}\right)$$

where σ represents the sigmoid activation function; ψ1, ψ2, and ψ3 denote the standard two-dimensional convolutional layers defined by kernel size k×k in all the branches of triplet attention [23]. Equation (2) can be simplified further as follows:

(3) $$S=\frac{1}{3}\left(\bar{\hat{T_{1}}\omega_{1}}+\bar{\hat{T_{2}}\omega_{2}}+X\omega_{3}\right)=\frac{1}{3}\left(\bar{S_{1}}+\bar{S_{2}}+S_{3}\right)$$

where $\omega_{1}$, $\omega_{2}$, and $\omega_{3}$ are the three cross-dimensional attention weights computed in triplet attention. The $\bar{S_{1}}$ and $\bar{S_{2}}$ in Equation (3) stands for the clockwise rotation operation which is performed to retain the initial input shape of (C×H×W).

**Figure 2:**
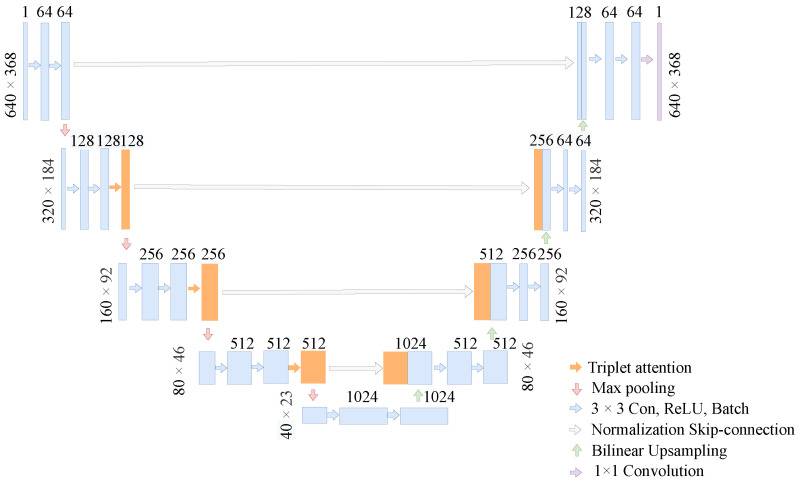
Illustration of the proposed TA-Unet. The model receives a sample size of 640 × 368 pixels as an input. Each blue arrow represents convolution operations with a 3 × 3 convolutional kernel followed by ReLU nonlinearity and batch normalization, the orange arrows represent triplet attention, and the red and green arrows stand for max-pooling and upsampling operations, respectively. The gray arrows connect the output of encoder layers with the input of corresponding decoder layers. The purple box in the decoder layer is the final segmentation map of the model.

The TA-Unet is a novel U-shaped framework based on the U-Net architecture. The model is composed of four encoding and decoding stages, and skip connections that allow to convey the low-level spatial information of the encoder to high-level layers of the decoder (see Figure 2). The only modification that we have introduced into our new TA-Unet architecture is that we have injected the attention mechanism into the encoder. Specifically, the triplet attention operation is performed after the first two cascaded convolution operations of the encoder stages. However, the first stage of the encoder remains unchanged, as in the original U-Net, as we do not want to focus on the noise too early. Adding attention mechanism too early would deteriorate the performance of the model. The resolution of the input image is 640×368, and the encoder layer, also known as the contracting path, is a series of operations consisting of convolution, max-pooling, and triplet attention mechanisms. The encoder layer consists of four blocks, each of which include two convolutions, one triplet attention, and one max-pooling operation, respectively, except the first block that does not include the attention mechanism, as mentioned above. The number of channels of the feature map is multiplied by two after each max-pooling operation. The size of the feature maps changes as shown in Figure 2, and the final feature map shape of the encoder is 40×23×512. Regarding the decoder layer, also known as expansive path, each block starts with upsampling the feature maps by two through deconvolution operation and halving channel size. Next, the resultant output is processed through two cascaded 3×3 convolutions and ReLU activation function after being concatenated with the output of the mirroring block of the contracting path. Finally, a 1×1 convolution operation is performed to extract the binary segmentation map.

## 4. Experiments and Discussion

In this section, we first introduce the dataset and metrics exploited in our experiments. Furthermore, we provide numerical results of our method and compare them to two previously proposed state-of-the-art methods. Finally, to further validate the efficiency of our method, we present visual segmentation maps of the proposed method as well as baselines on samples that were taken at different times of the day and in varying weather conditions.

### 4.1. Datasets

In order to demonstrate the efficiency and the performance of the proposed model, we adopt the publicly available UESTC ALL-Day Scenery (UAS) dataset provided by the University of Electronic Science and Technology of China [31]. The dataset consists of a total of 6380 images taken at varying day times and in varying weather conditions. It includes 1995 samples taken in the sunshine, 2167 samples taken at night, 819 samples taken in the rainy condition, and 1399 samples taken at dusk. We name these four sets as sun set, night set, rain set, and dusk set for the sake of better representation. The resolution of all images is 640×360, and we resize them to 640×368 before feeding into the network.

### 4.2. Implementation Details

For model optimization, we use the Adam algorithm, and the initial learning rate is set to 0.0005 [32]. Cross entropy (Equation (4)) is a common loss function used in segmentation tasks to deal with a binary classification task, which calculates the probability of belonging to one class or to the other [33]. However, it simply represents the error for each pixel without giving importance to the particular class that one focuses on. In our drivable road region segmentation task, the road edge area needs more focus. Thus, using only one loss function is not enough to attain quality results. The Lovasz–Softmax loss function (Equation (8)), which is the optimization of the evaluation metric IoU, is designed specifically for segmentation tasks [34]. In this paper, we adopt a loss function which is the combination of cross-entropy loss ($L_{\left(Cross-entropy\right)}$) and Lovasz-Softmax loss ($L_{\left(Lovasz-Softmax\right)}$) (Equation (10)), and it can be demonstrated as follows:

(4) $$L_{\left(Cross-entropy\right)}=-\frac{1}{p}\sum_{i=1}^{p}logf_{i}\left(y{{}^{*}}_{i}\right)$$

(5) $$f_{i}\left(c\right)=\frac{e_{i}^{F}\left(c\right)}{\sum_{c}^{f}\in ce_{i}^{F}\left(c^{i}\right)},i\in \left[1,p\right],c\in C$$

(6) $${\tilde{y}}_{i}=argmaxF_{i}\left(c\right)$$

(7) $$J_{c}\left(y^{*},\tilde{y}\right)=\frac{|\left(y^{*}=c\right)\cap \left(\tilde{y}=c\right)|}{\left|(\left(y^{*}=c\right)\cup \left(\tilde{y}=c\right)\right|}$$

(8) $$L_{Lovasz-Softmax}=\Delta J_{c}\left(y^{*},\tilde{y}\right)=1-J_{c}\left(y^{*},\tilde{y}\right)$$

(9) a+b=1

(10) $$L=aL_{Lovasz-Softmax}+bL_{\left(Cross-entropy\right)}$$

### 4.3. Evaluation Metrics

For a comprehensive comparison, we adopt three metrics to evaluate the segmentation models on our dataset, and they are pixel accuracy (Acc), mean intersection of union (mIoU), and parameter size of models. One of the straightforward ways to measure the performance of a semantic segmentation model is to calculate the proportion of correctly classified pixels out of all the pixels in an image, and it is called pixel accuracy. In practice, we can see both conditions where pixel accuracy is calculated for each class individually, or for all classes globally at the same time. On the other hand, mIoU, also known as Jaccard index, highlights the intersection of the predicted segmentation map and the ground truth divided by the union of them. To obtain the final results of mIoU, we first calculate mIoU for each class and then take the mean average of them. The mathematical expressions of Acc and mIoU are as follows:

(11) $$Acc=\frac{TP+TN}{TP+TN+FP+FN}$$

(12) $$mIoU=\frac{TP}{TP+FP+FN}$$

where TP stands for true positive predictions, TN represents true negative predictions, FP denotes false positive predictions, and FN indicates false negative predictions.

### 4.4. Results and Analysis

Table 1 compares the mIoU results of our model TA-Unet to the framework proposed in the UAS dataset paper, titled as SGSN across four image sets, and also all sets together. As is evident from the table, TA-Unet negligibly improves the mIoU results for the dusk set, night set, and sun set. A huge improvement was detected in the rain set and also when all the sets are trained together, where the proposed model achieved 98.03% and 97.41%, respectively, with around 1% improvement from the baseline SGSN framework in both cases.

**Figure 3:**
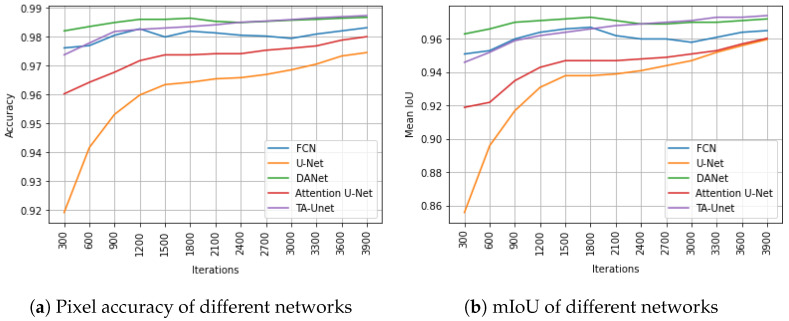
Pixel accuracy and mIoU of different networks on the validation set. The *x*-axis represents pixel accuracy (PA) and mean IoU in subfigures (**a**,**b**), respectively, while the *y*-axis stands for number of iterations in both subfigures.

To further validate the efficiency and performance of the proposed model, we compare the results to four state-of-the-art deep-learning-based models’ results, i.e., fully convolutional networks for segmentation (FCN), dual-attention network for scene segmentation (DANet), U-Net, and Attention U-Net. For a fair comparison, all the baselines were trained using the same training hyperparameters on the same hardware platform. Figure 3 reveals the learning curves of the proposed model and the baselines during the validation. Specifically, Figure 3a portrays the pixel accuracy for the models, and Figure 3b highlights the mIoU results.

As can be noted from Figure 3, the proposed model dominates in terms of both pixel accuracy and mIoU metrics. TA-Unet starts from over 97% and 94% pixel accuracy and mIoU, respectively, and hits the 98% and 96% benchmark score after 1000 iterations. The final pixel accuracy and mIoU scores of TA-Unet are 98.74% and 97.41%, respectively (see Table 2). Among the baselines, DANet yields the most promising results on the exploited dataset. Among all models, U-Net experiences slower convergence, starting from 92% and 86% pixel accuracy and mIoU, respectively. However, at the end of the training, the results of U-Net level off with Attention U-Net in terms of mIoU and shrink the gap in the pixel accuracy score up to less than 1%. Although FCN and DANet networks performed well in the beginning of the training process, TA-Unet outperformed them as the iteration progressed.

As is mentioned above, the UAS dataset suffers from a class-imbalance problem. Nowadays, class-imbalanced image segmentation is a very hot topic in the research, and adopting more than one loss function is one of the common solutions to overcome the problem. The positive effect of mix loss function on performance has been successfully proven in several papers [35,36]. With the same aim, we adopt mix loss function on the backbone of TA-Unet, and compare its results with the model trained on a single loss function, as shown in Table 3. The results confirm that the mix loss function boosts the performance of the model in terms of both pixel accuracy and mean intersection over union.

**Figure 4:**
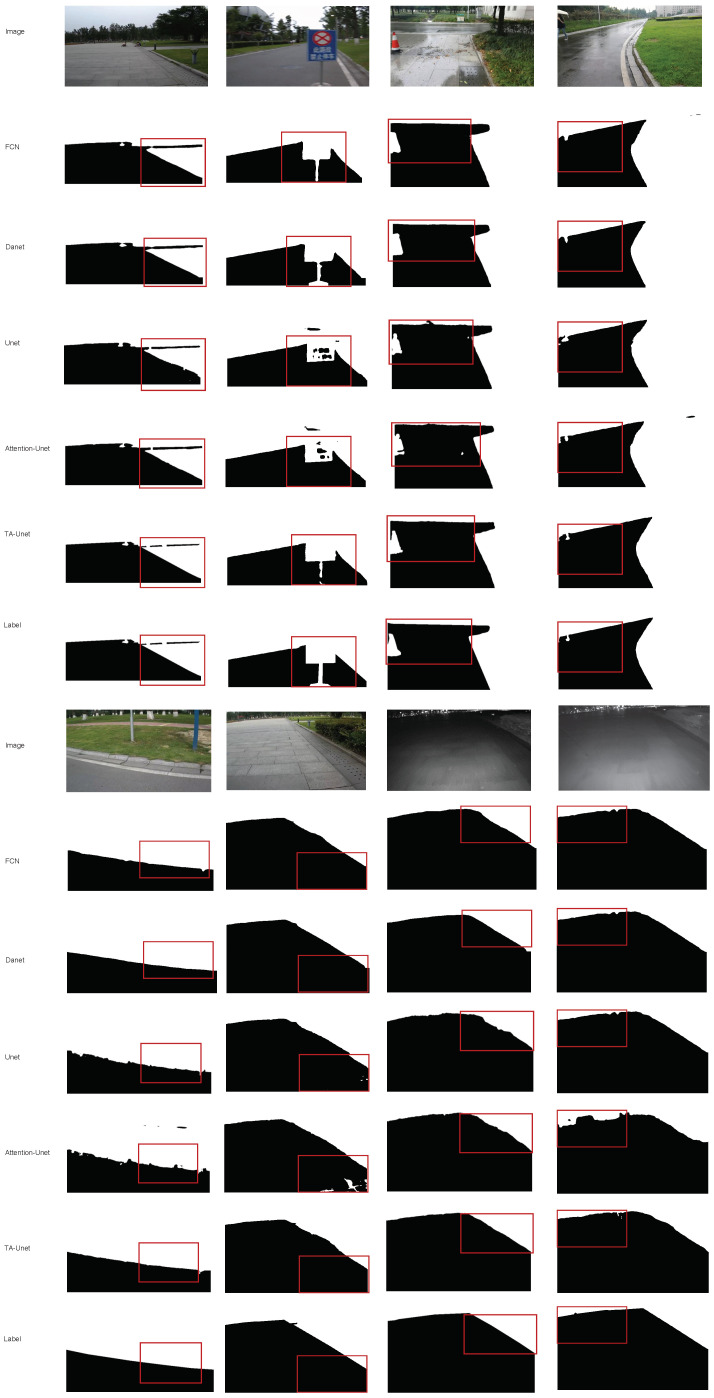
Road segmentation results of different methods in different conditions.

The results of extensive experiments conducted by us demonstrate that the TA-Unet demonstrates consistently better performance than the baselines. Additionally, Figure 4 highlights some of the road segmentation results of all methods at different day times and under varying weather conditions. As is visible from the figures, the proposed method yields clearer segmentation maps compared to other methods.

## 5. Conclusions

In this work, we have proposed a novel architecture, dubbed TA-Unet, which incorporates triplet attention mechanism into the U-Net-like architecture to effectively extract road segmentation maps. Specifically, we placed the attention mechanism after the convolution operations at each stage of the encoder model to preprocess the output feature maps of each stage before concatenating them with the mirroring decoder stage inputs. Triplet attention is a powerful attention module which captures important features across dimensions and is calculated through channel attention and spatial attention. To validate the efficiency and the performance of the proposed model, we adopted the UAS dataset that includes images captured at varying times of the day and in varying weather conditions. The extensive experiments demonstrate that the proposed model outperforms baseline networks in terms of metrics such as pixel accuracy and mean intersection over union. On top of that, TA-Unet produces relatively clearer segmentation maps under different weather conditions. Furthermore, adopting mix loss functions can lead to a boost in the performance.

Although the parameter size of the network is smaller than the baselines, it is still computationally expensive for real-time segmentation. We believe that there is still a lot of room for improvement in terms of inference time speed and accuracy. In the future, we intend to continue our research in the following aspects: 1. Utilizing datasets of complex environments, such as curves under complex road conditions, road conditions during snowy days, etc., in order to improve the learning ability of the network in complex environments. 2. Scene expansion. The dataset exploited in this paper includes images captured in urban road sections. In the future, we will work on datasets that include samples taken in rural road sections, mountainous roads, etc., which can simulate more realistic environments. 3. Designing lightweight networks for real-time segmentation. 

## Figures and Tables

**Table 1:** The mIoU scores of the proposed TA-Unet and SCGN framework on the UAS dataset.

Dataset	SGSN	TA-Unet
Dusk set	98.04	98.18
Night set	94.01	94.39
Rain set	97.04	98.03
Sun set	97.58	97.85
UAS	96.40	97.41

**Table 2:** Quantitative results.

Method	Accuracy	mIoU	Parameters
FCN	98.32	96.50	97.25 M
U-Net	97.46	95.97	13.40 M
DANet	98.68	97.20	47.51 M
Attention U-Net	98.01	96.04	34.89 M
TA-Unet	98.74	97.40	31.05 M

**Table 3:** Performance of TA-Unet when trained on different loss functions.

	Cross-Entropy Loss Function	Lovasz-Softmax Loss Function	Mixed Loss Function
acc	98.66	98.68	98.74
mIoU	97.29	97.30	97.41

## Data Availability

Not application.
